# Lengthened Cutaneous Silent Period in Fibromyalgia Suggesting Central Sensitization as a Pathogenesis

**DOI:** 10.1371/journal.pone.0149248

**Published:** 2016-02-12

**Authors:** Seol-Hee Baek, Hung Youl Seok, Yong Seo Koo, Byung-Jo Kim

**Affiliations:** Department of Neurology, Korea University Medical Center, Korea University College of Medicine, Seoul, Korea; Charité University Medicine Berlin, GERMANY

## Abstract

The pathogenesis of fibromyalgia (FM) has not been clearly elucidated, but central sensitization, which plays an important role in the development of neuropathic pain, is considered to be the main mechanism. The cutaneous silent period (CSP), which is a spinal reflex mediated by A-delta cutaneous afferents, is useful for the evaluation of sensorimotor integration at the spinal and supraspinal levels. To understand the pathophysiology of FM, we compared CSP patterns between patients with FM and normal healthy subjects. Twenty-four patients with FM diagnosed in accordance with the 1990 American College of Rheumatology classification system and 24 age- and sex-matched healthy volunteers were recruited. The CSP was measured from the abductor pollicis brevis muscle. Demographic data, number of tender points, and visual analog scale and FM impact questionnaire scores were collected. The measured CSP and clinical parameters of the patient and control groups were compared. In addition, possible correlations between the CSP parameters and the other clinical characteristics were analyzed. Mean CSP latencies did not differ between patients (55.50 ± 10.97 ms) and healthy controls (60.23 ± 11.87 ms; p = 0.158), although the mean CSP duration was significantly longer in patients (73.75 ± 15.67 ms) than in controls (63.50 ± 14.05 ms; p = 0.021). CSP variables did not correlate with any clinical variables. The significantly longer CSP duration in FM patients suggests central dysregulation at the spinal and supraspinal levels, rather than peripheral small fiber dysfunction.

## Introduction

Fibromyalgia (FM) is a syndrome characterized by chronic widespread pain and variable symptoms such as sleep disturbances, fatigue, depression, and cognitive dysfunction[1]. The mechanism underlying chronic pain in FM remains unclear, but cumulative evidence has suggested that central pain amplification plays a key role in the fundamental pathogenesis of FM. Pain amplification refers to augmented pain and sensory processing within the spinal cord and brain, and is sometimes termed “central sensitization”[2]. Recent neuroimaging studies have contributed greatly to our understanding of functional and morphological changes of the brain structures involved in pain systems in FM. A functional magnetic resonance imaging (fMRI) study showed that, in response to painful stimuli, FM patients have increased neuronal activation in pain-related brain regions, including the primary and secondary somatosensory cortex, insula, and anterior cingulate cortex[3]. Voxel-based morphometry (VBM) has demonstrated reduced gray matter volumes in 3 regions: the anterior, mid-cingulate, and mid-insular cortices[4]. In addition to a number of neuroimaging studies, various electrophysiological tools such as nociceptive flexion reflex and transcranial magnetic stimulation have been used to examine central pain processing in patients with FM. Nociceptive flexion reflex studies in FM show a decreased pain threshold in patients with FM compared with healthy individuals[5, 6]. Furthermore, two recent studies using transcranial magnetic stimulation have reported decreased intracortical inhibition in patients with FM, suggesting the presence of deficient central inhibitory mechanisms[7, 8].

The cutaneous silent period (CSP) is a useful tool for investigating pain processing in both the central and peripheral nervous systems. The CSP is a brief pause in muscle action potentials following strong stimulation of the cutaneous nerve during a sustained voluntary contraction[9], and is considered a protective reflex mediated by the spinal inhibitory circuit and reinforced by parallel modulation of the motor cortex[10]. To our knowledge, only two studies have used the CSP to investigate the processes underlying the pain in FM[11, 12]. These studies showed significantly prolonged CSP onset latencies in patients with FM compared to controls, but did not report any differences in CSP duration between groups. On the basis of these results, the pain mechanism in FM is thought to be associated with a dysfunction of A-delta fibers, which is in contrast to previous work suggesting a pathophysiological role of the central nervous system (CNS) in FM. Since FM is diagnosed based on certain criteria and the exclusion of other possible causes, these discrepancies might have resulted from methodologic flaws related to the patient inclusion criteria. In both studies, patients were grouped according to symptoms and nerve conduction study (NCS) findings, and thus FM patients in those two studies may have included patients with small fiber neuropathies (SFNs).

Based on the assumption that central sensitization is the main mechanism of pain processing in FM, this study compared the CSP in patients with FM confirmed not to have peripheral nerve dysfunction including small fiber through assessment using NCSs, needle electromyography (EMG), and the quantitative sudomotor axonal reflex test (QSART), with that in healthy control subjects.

## Materials and Methods

### Subjects

Patients with FM were recruited from the neurology clinic at a university-affiliated hospital from September 2013 to October 2014. FM was diagnosed by history-taking and clinical assessment according to the 1990 American College of Rheumatology criteria for FM[13]. Any patient who had a history of distal symmetric paresthesia or who exhibited abnormal sensory examination results (pinprick and thermal sensory loss) indicative of small fiber dysfunction was initially excluded. Patients were also excluded if they had a history of any specific muscle disease, neuromuscular junction disorder, spinal surgery, or medical condition associated with peripheral neuropathy such as diabetes mellitus, alcohol abuse, metabolic disorders, malignancy, or long-term drug use. All participants were given the NCS/EMG and QSART, and patients who had any abnormal findings were excluded. Twenty-four healthy volunteers without any neurological or medical problems were recruited as control subjects.

### Ethics statement

Written informed consent was obtained from all participants before study inclusion. All procedures were in accordance with the Declaration of Helsinki and approved by the Korea University Medical Center Institutional Review Board (IRB no. ED13139).

### Clinical evaluation for FM

Demographic data, including age, sex, height and weight, medical history, and disease duration were recorded. The severity of pain was assessed using a visual analog scale (VAS) of 0 to 100 and tender point count. The Korean version of the FM impact questionnaire (K-FIQ), which consists of 10 items, was used to assess disease severity, functional disability, and quality of life[14].

### Measurement of CSP and other electrodiagnostic studies

NCS and CSP measurements were performed using standard electrodiagnostic equipment (Viking Select; Viasys Healthcare, Madison, WI, USA). During NCS and measurement of CSP parameters, each patient’s skin temperature on the dorsum of the hands and feet was confirmed to be ≥32°C. NCS was performed using a 1-cm-diameter, disposable, flat-surface electrode in both upper and lower extremities of all patients and healthy controls. Motor NCSs were performed by stimulating the median, ulnar, peroneal, and posterior tibial nerves at the wrist, ankle, and knee, with recording at the abductor pollicis brevis (APB), abductor digiti minimi, extensor digitorum brevis, and abductor hallucis. Sensory NCSs were performed with median, ulnar, superficial peroneal and sural nerve stimulation. F-waves and H-reflexes were measured. To minimize the effects of confounding variables, NCS performance was controlled and regulated in a standard electrodiagnostic laboratory environment[15].

**Fig 1:**
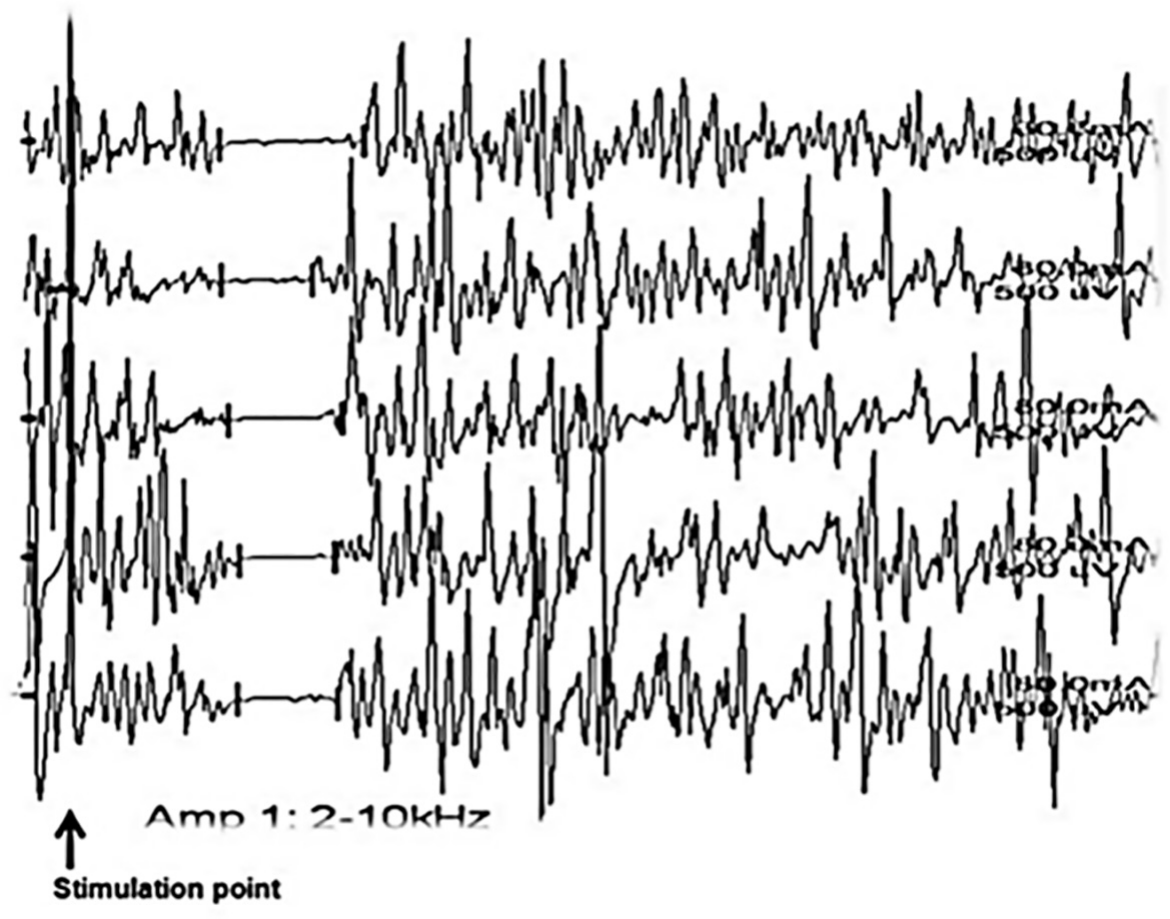
An example of CSP waves in a subject. The raw EMG signal was recorded from the APB muscle after stimulation of the index finger at an 80-mA intensity. We repeatedly measured the CSP waves a minimum of 20 times. Five traces with a complete silent period were selected.

The CSP was recorded by an electromyographer using previously published methodology[10, 16, 17]. Ring electrodes were used to stimulate the index finger. Electromyographic activity was recorded using surface electrodes from the APB, with a filter setting of 2 Hz–10 kHz. To obtain steady maximal contraction, the subject was asked to contract against resistance, and an EMG audiosignal was used to monitor muscle contraction. During maximal voluntary contraction, a single painful stimulus (80-mA intensity) with a 0.5-ms duration was delivered to the index finger until a complete silent period of reproducible latency and duration was obtained. Before measurement, we provided the subject with instructions, and then repeated the test at least 20 times until we obtained five optimal recordings showing complete silencing of the motor unit potential with the longest duration and shortest latency (Fig 1). To decrease possible variation in CSP parameters, we used the mean value of the five best CSPs as the final value for CSP parameters in each subject. CSP latency was defined as the time between the stimulation and beginning of the silent period. CSP duration was defined as the time between the beginning and endpoint of the silent period. The beginning and endpoint latencies of each CSP were identified by visual inspection at the beginning of an abrupt decrease or upon return of EMG activity. The latencies, durations, and endpoints of CSPs were used in the final analysis. In addition, we rectified each of the CSP traces to reduce artifacts and analyzed the mean CSP parameters of longest duration and shortest latency.

To evaluate SFN according to the Herrmann diagnostic criteria[18], QSART was performed using an automatic function test system (Q-Sweat; WR Medical Electronics, Maplewood, MN, USA), which was controlled and regulated by the standards of an electrodiagnostic laboratory environment[19]. The stimulus consisted of 10% iontophoresed acetylcholine with a constant current generator at 2 mA for 5 minutes. Sweat volumes were recorded in the central compartment of a multicompartmental sweat cell from the following 4 sites: the proximal forearm (25% of the distance from the ulnar epicondyle to the pisiform bone), distal forearm (75% of the distance from the ulnar epicondyle to the pisiform bone), proximal leg (5 cm distal to the fibular head), and distal leg (5 cm proximal to the medial malleolus). The QSART results were considered abnormal if sweat volume was lower than age- and sex-specific reference values.

### Statistical analysis

Comparisons of CSP parameters and demographic data between patients with FM and control subjects were performed using the independent *t*-test (for CSP parameters, age, and height) or the chi-square test (for gender). Pearson correlation was used to analyze correlations between the CSP parameters and clinical data, such as VAS score, K-FIQ score, age, and height. Paired t-tests were used to analyze the differences between CSP parameters measured by visual inspection and by rectification. Statistical analyses were performed using the Statistical Package for the Social Sciences (SPSS; release 22.0; Chicago, IL, USA), and *p-*values <0.05 were considered to indicate a statistically significant difference.

## Results

### Clinical characteristics of the subjects

Data for 24 patients with FM and 24 healthy subjects were included in the analysis. Age, height, CSP onset latency, and CSP duration followed a normal distribution in both patient and control groups.

Women composed 95.8% (23/24) of the patient group and 87.5% (21/24) of the control group. The mean age for the patient and control groups was 45.2 years (range 20–72 years) and 48.5 years (range 21–69years), respectively. The mean height for the patient and control group was 159.3 cm and 157.4 cm, respectively. There were no significant differences in age, sex, or height between the two groups (Table 1).

**Table 1:** Demographic data of patients with fibromyalgia and controls.

	Patient group (n = 24)	Control group (n = 24)	p-value
**Sex (female, %)**	23 (95.8%)	21 (87.5%)	0.609
**Age (years)**	45.21 ± 14.38	48.54 ± 11.84	0.385
**Height (cm)**	159.25 ± 5.75	157.42 ± 6.14	0.291
**TPC (range)**	16.9 (11–18)	-	
**VAS (range)**	63.8 (50–80)	-	
**FIQ (range)**	60.7 (32–91)	-	

TPC, tender point count; VAS, visual analog scale; FIQ, fibromyalgia impact questionnaire.

Data are presented as mean ± standard deviation. A p-value < 0.05 is considered significant

### Comparison of CSP parameters between the patient and control groups

CSP onset latencies did not differ significantly between the patient (55.50 ± 10.97 ms) and control groups (60.23 ± 11.87 ms; p = 0.158). However, CSP durations in the patient group (73.75 ± 15.67 ms) were significantly longer than those in the control group (63.50 ± 14.05 ms; p = 0.021) (Table 2). CSP parameters did not correlate with clinical parameters, such as VAS score, K-FIQ score, age, or height.

**Table 2:** The onset latency and duration of patients with fibromyalgia and controls.

	Patient group(n = 24)	Control group(n = 24)	p-value
**CSP latency (msec)**	55.50 ± 10.97	60.23 ± 11.87	0.158
**CSP duration (msec)**	73.75 ± 15.67	63.50 ± 14.05	0.021

CSP, cutaneous silent period.

Data are presented as mean ± standard deviation. A p-value < 0.05 is considered significant

### Comparison of CSP parameters between raw EMG data and rectified data

There were no significant differences in the mean CSP onset latency and mean CSP duration between raw EMG data and rectified data (p = 0.218 and p = 0.379, respectively).

## Discussion

Mean CSP duration was significantly prolonged in our patients with FM. The circuitry for CSP is configured such that afferent limbs of the CSP derive from A-delta fibers and the motor neurons receive postsynaptic inhibition through spinal inhibitory interneurons during the CSP[9]. Although the central processing in the CSP circuitry is not fully understood, the CSP might be modulated by the descending fiber from the motor cortex to the spinal cord[10]. Several studies have investigated the CSP in patients with central nervous disorders. Pullman et al. reported that CSP durations were significantly longer in patients with brachial dystonia than in controls, while CSP onset latencies did not differ between groups[20]. The prolonged duration of CSP in patients with Parkinson’s disease was significantly lower after l-dopa treatment[21]. Patients with multiple system atrophy also show prolonged CSP duration[22]. In addition, Han et al. found significantly longer CSP duration in patients with restless legs syndrome (RLS), which is considered a central sensitization syndrome[10]. These previous studies suggest that CSP duration may reflect dysfunction of supraspinal control, which exerts modulatory influences on spinal excitability. Therefore, our results support the notion that FM is associated with dysfunction of pain modulation mechanisms in the CNS.

We found that the mean CSP latency in patients with FM did not differ from that in healthy controls. CSP onset latency might be determined by three segments involving different parts of the circuitry: the peripheral conduction time from stimulation site to spinal cord via A-delta fibers, the central time required for processing inhibition, and the efferent time from the spinal cord to muscle motor fibers[23, 24]. The delayed onset latency of CSP has been attributed primarily to a long afferent conduction time, rather than a long central delay[23]. Many clinical studies have demonstrated a delayed CSP latency in patients with peripheral neuropathies, suggesting that dysfunction of afferent A-delta fibers is responsible for prolonged CSP latency. Patients with diabetes with small fiber neuropathy experience a significant delay in CSP latency[17, 25, 26]. A prolonged CSP latency is also observed in patients with HIV-related peripheral neuropathies[27]. In addition, studies of entrapment neuropathies such as carpal tunnel syndrome have reported significantly prolonged CSP latency in patients compared to healthy control subjects[16, 28]. These previous studies indicate that CSP onset latency could be primarily affected by afferent impulses from A-delta fibers rather than by supraspinal control in the CNS. In contrast, we found no significant differences in CSP latency between FM patients and controls, and suggest that FM may not be associated with dysfunction of afferent A-delta fibers.

Converging evidence from functional neuroimaging studies has provided a robust basis for the hypothesis of central pain amplification in the fundamental pathogenesis of FM. A single-photon emission computed tomography study showed decreased regional cerebral blood flow in the thalamus and caudate nucleus in 10 patients with FM, suggesting that abnormal pain perception in FM is associated with functional abnormality within the CNS[29]. Two recent studies using fMRI have also shown increased brain activation within pain-related regions[3], and decreased connectivity within the descending pain inhibitory network in FM[30]. In a VBM study of 10 patients, Kuchinad et al. demonstrated reduced gray matter density in the cingulate, insular, and medial frontal cortices, and parahippocampal gyrus[31]. In addition to gray matter abnormalities, a recent study using diffusion tensor imaging demonstrated increased fractional anisotropy in the postcentral gyri, amygdalae, hippocampi, superior frontal gyri, and anterior cingulate gyri of 30 FM patients, reflecting microstructural white matter changes of brain areas related to endogenous pain inhibition[32]. Cerebrospinal fluid biochemical studies also support the hypothesis of central pain amplification in FM. Cerebrospinal fluid of patients with FM contains higher levels of substance P and glutamate[33, 34], but lower levels of serotonin, norepinephrine, and dopamine, compared to controls[35]. Either increased levels of neurotransmitters that facilitate pain transmission or decreased levels of neurotransmitters that inhibit nociceptive stimulation could augment central pain processing[36]. Although the pathophysiology of FM is not yet fully elucidated, central sensitization is considered the main pathogenesis of FM based on previous studies. Our results further support this hypothesis.

To the best of our knowledge, only two studies have previously investigated the CSP in FM, comparing CSP parameters to controls in 28[11] and 32[12] patients and controls, respectively[11, 12]. In contrast to our findings, both observed longer CSP latency, but no difference in CSP duration, in patients compared to healthy normal subjects. This discrepancy may be related to the patient inclusion criteria. SFN is a well-known cause of chronic symmetric pain and often remains undiagnosed because its symptoms are wide-ranging, largely non-specific, and not detected by diagnostic testing using NCSs[37]. Quantitative sensory testing, skin biopsy and quantification of intradermal nerve fiber density, sympathetic skin response or QSART are used for evaluation of SFN[37–39]. We excluded all patients with indications of SFN based on clinical features and QSART findings. SFN and FM share clinical characteristics, including not only chronic pain, but also fatigue, headache, and gastrointestinal symptoms. Many patients with SFN are diagnosed with FM before a final diagnosis of SFN is reached. One study identified SFN in 59% of patients with childhood-onset chronic widespread pain who were initially diagnosed with FM[40].

Recently, several studies have suggested that SFN could be the cause of, or at least a contributing factor to, pain in FM. Some studies using skin biopsy showed decreased intraepidermal nerve fiber density in patients with FM[41–45]. Impaired quantitative sensory testing and pain-related evoked potentials were also observed in FM patients[41]. In addition, a corneal confocal bio-microscopy study revealed that 17 FM patients have thinner corneal stromal nerves and diminished sub-basal plexus nerve density[46]. Although recent findings suggested the potential role of small fibers in FM, small fiber involvement seems to be limited to a subset of patients with FM. In previous studies using skin biopsy, a minority of FM patients (11 of 27 patients in Oaklander’s study, 6 of 20 patients in Giannoccaro’s study, and 15 of 46 patients in Kosmidis’s study) had diminished intraepidermal nerve fiber density[43–45]. In addition, Uceyler et al. recently suggested that it is essential to distinguish between SFN and small fiber pathology[47]. Small fiber lesions can be found not only in SFN, but also in various diseases such as RLS and complex regional pain syndrome[48, 49]. Although some patients with FM have small fiber lesions, whether small fiber pathology is pathogenic or represents a secondary change in FM remains controversial. It is also possible that SFN represents a comorbid disease in some cases of FM. Future study is needed to clarify these relationships.

In our patients, CSP parameters did not correlate with clinical parameters such as VAS score, K-FIQ score, age, and height. It remains controversial whether the CSP is reliable for evaluating disease severity. Umay et al. reported that prolonged CSP latency in the lower extremities correlated with FIQ score and the physical health subscale of Short Form-36, although there was no correlation between CSP latency in the upper extremities and other clinical parameters[12]. However, most studies have not suggested the possibility that CSP could be useful for evaluating pain severity. Koo et al. found no statistically significant correlation between CSP parameters and scores on clinical symptom questionnaires in patients with carpal tunnel syndrome[16]. Han et al. reported that CSP parameters of patients with RLS did not correlate with clinical variables, including the International Restless Leg Severity Scale score[10]. Inghilleri et al. reported no changes in CSP parameters after administration of fentanyl in healthy subjects, even though the painful sensation evoked by electrical stimulation was significantly decreased[50]. Given these results, CSP parameters appear not to be correlated with pain severity. Further studies with larger numbers of subjects will be needed to investigate the relationship between CSP parameters and clinical data.

This study was limited by the small number of subjects. Because the measured CSP values were inconsistent on repeated measurement, a larger number of subjects are needed to reduce random error. To overcome this limitation, we measured CSPs repeatedly, at least 20 times in each subject. The CSP measurements were obtained by an expert electromyographer who carefully selected five traces with a complete silent period.

In conclusion, dysfunction of supraspinal control may be responsible for pain in FM, providing further evidence that central sensitization underlies the pathogenesis of FM.
